# Ionizing radiation and Alzheimer’s disease: epidemiology, mechanisms, and eCIRP-mediated tau pathology

**DOI:** 10.3389/fimmu.2026.1961728

**Published:** 2026-09-22

**Authors:** Max Brenner, Archna Sharma, Ping Wang

**Affiliations:** 1 Center for Immunology and Inflammation, The Feinstein Institutes for Medical Research, Manhasset, NY, United States; 2 Department of Surgery, Zucker School of Medicine at Hofstra/Northwell, Manhasset, NY, United States; 3 Department of Molecular Medicine, Zucker School of Medicine at Hofstra/Northwell, Manhasset, NY, United States

**Keywords:** Alzheimer’s disease, dementia, extracellular cold-inducible RNA-binding protein, ionizing radiation, neuroinflammation, tauopathy

## Abstract

As human radiation exposure expands through medical imaging, nuclear industry growth, and the acceleration of crewed space exploration, understanding the molecular mechanisms linking radiation to Alzheimer’s disease (AD) is becoming an increasingly urgent priority. Here, we review epidemiological, mechanistic, and therapeutic evidence for a relationship between ionizing radiation and AD, focusing on a neuroinflammatory pathway centered on extracellular cold-inducible RNA-binding protein (eCIRP). Epidemiological support comes from occupational cohorts of nuclear workers and studies linking residential radon to AD mortality; a meta-analysis of 18 studies finds an 11% increased all-cause dementia risk per 100 mSv. Mechanistically, ionizing radiation generates DNA double-strand breaks and reactive oxygen species that activate the ataxia-telangiectasia mutated (ATM) kinase and trigger rapid microglial activation, and causes the nuclear-to-cytoplasmic translocation and extracellular release of the danger-associated molecular pattern eCIRP. Released eCIRP activates neuronal cyclin-dependent kinase 5 (Cdk5), resulting in AD-like tau phosphorylation. This pathway is validated by loss-of-function studies in CIRP-knockout mice and pharmacological blockade with the eCIRP inhibitor C23. Beyond tau phosphorylation, eCIRP drives mitochondrial dysfunction and ferroptosis in myeloid cells and activates endothelial cell death pathways that may compromise blood–brain barrier integrity. eCIRP links radiation-induced neuroinflammation to tau-driven neurodegeneration, and eCIRP inhibitors warrant investigation for both radiation-induced cognitive dysfunction and sporadic AD, where eCIRP is similarly elevated. Translating these mechanistic insights into eCIRP-targeted therapies and validated biomarkers could open a new front in the prevention and treatment of these prevalent and debilitating disorders.

## 1 Introduction

Dementia and Alzheimer’s disease (AD) affect over 55 million people worldwide, and the number in the USA alone is projected to reach 13.8 million, representing 3.8% of the population by 2060 (1). Despite decades of research and the development of disease-modifying therapies targeting amyloid-β and tau pathology, effective preventive and therapeutic approaches remain elusive. Genetic factors, particularly apolipoprotein E (APOE) variants, account for 60%–80% of AD risk in twin studies, but environmental factors are gaining attention as modifiable contributors to disease development (2–7). Among these, ionizing radiation stands out as both widespread and understudied (8–10).

High-energy radiation—so called because it carries enough energy to eject electrons from atoms producing charged ions that damage biological molecules—encompasses electromagnetic radiation (X-rays and gamma rays) and particulate radiation (α particles, β particles, protons, neutrons, and heavy ions such as ^56^Fe) (11). Radiation exposure is quantified in several related but distinct units. The absorbed dose, measured in grays (Gy; 1 Gy = 1 J/kg), describes the energy deposited per unit mass of tissue. The equivalent dose, measured in sieverts (Sv), adjusts the absorbed dose by a radiation weighting factor that accounts for the biological effectiveness of different radiation types: for X-ray and gamma-ray photons, the weighting factor is 1 (1 Gy = 1 Sv), whereas for alpha particles, it is 20 (1 Gy = 20 Sv). The effective dose, also in sieverts, further incorporates tissue weighting factors to estimate whole-body stochastic risk from non-uniform exposures and is therefore not equivalent to the absorbed dose to any single organ (11). The millisievert (mSv) is the standard unit for occupational and environmental exposures. In this review, low-dose ionizing radiation (LDIR) refers to exposures below approximately 0.1 Gy, typical of occupational and environmental settings. Studies in Section 3.3 that deliberately apply radiation as a therapeutic intervention in AD models use doses ranging from mGy to several Gy—low relative to conventional cancer radiotherapy (40–70 Gy) but far above LDIR. Absolute dose values are specified throughout to avoid ambiguity.

Human exposure to ionizing radiation is remarkably common. Medical imaging is the most frequent source: a single head computed tomography (CT) is associated with an estimated effective dose of approximately 2 mSv (actual doses vary with scanner model, protocol, and patient anatomy), and the average American receives approximately 3 mSv per year from medical procedures alone (12). Occupationally, nuclear facility workers, uranium processors, interventional radiologists, and cardiac catheterization laboratory personnel accumulate chronic exposures over careers spanning decades (13–16). Environmentally, residential radon (a radioactive gas that seeps from soil into buildings) is the largest natural source of ionizing radiation and exposes virtually the entire population (8, 9). Cosmic radiation poses a distinct and growing concern. At commercial aviation altitudes, aircrew receive 2–5 mSv per year, and military aviators flying at higher altitudes or in polar routes accumulate more (17). The risk escalates sharply for space travelers. Astronauts aboard the International Space Station receive roughly 150 mSv over a 6-month orbital mission, and the radiation environment beyond low Earth orbit is far harsher: NASA’s Artemis program aims to return humans to the lunar surface, where galactic cosmic radiation (GCR) and solar particle events are unshielded by Earth’s magnetosphere, and planned Mars transit missions would expose crews to an estimated 600–1,000 mSv over a 2.5-year round trip (18). Commercial deep-space ventures may place growing numbers of private citizens in this high-radiation environment (19, 20). The cognitive consequences of these exposures, particularly the potential for radiation to initiate or accelerate AD, are therefore relevant to an expanding population.

Neuropathologically, AD is characterized by two hallmark lesions: extracellular plaques containing aggregated amyloid-β and intracellular neurofibrillary tangles (NFTs) composed of hyperphosphorylated microtubule-associated protein tau (21, 22). While amyloid-β deposition initiates pathological cascades in early-stage AD, tau pathology—particularly the presence of paired helical filaments (PHF) and phosphorylated tau at pathological sites—shows the strongest correlation with neurodegeneration, cognitive deficits, and disease progression (23–27). Under normal conditions, tau stabilizes microtubules and supports axonal transport. Hyperphosphorylation at sites including Ser199, Ser202, Thr205, Ser208, Ser396, and Ser404 disrupts this function, causing tau to detach from microtubules, self-aggregate, and ultimately trigger neuronal death (22, 28, 29). The kinases that drive pathological tau phosphorylation are well characterized; Cdk5 and glycogen synthase kinase-3β (GSK3β) are the principal tau kinases in AD (29–33). Cdk5 activity is dramatically elevated in AD brain through mechanisms involving cleavage of the regulatory subunit p35 into a hyperactivating truncated form p25 by the calcium-dependent protease calpain, resulting in Cdk5-mediated tau hyperphosphorylation (32, 34).

Recent mechanistic investigations by our group have demonstrated that ionizing radiation causes AD-like tau phosphorylation through a neuroinflammatory pathway involving the danger-associated molecular pattern (DAMP) extracellular cold-inducible RNA-binding protein (eCIRP) (35). CIRP is constitutively expressed in the nucleus; under stress, it translocates to the cytoplasm and is released extracellularly, where it acts as a potent neuroinflammatory mediator (36–39). Since microglial cells release eCIRP in response to hypoxia, alcohol, and amyloid-β (38–40), this DAMP may serve as a common mechanism through which diverse stressors promote tau pathology and cognitive dysfunction (35, 40–43). The finding that the eCIRP inhibitor C23 blocks radiation-induced tau phosphorylation *in vivo* (35) raises the possibility that targeting eCIRP could benefit both radiation-exposed individuals and patients with sporadic AD.

In the sections that follow, we first review the epidemiological evidence linking radiation to dementia and then examine the mechanistic pathways—including oxidative stress, DNA damage, ataxia-telangiectasia mutated (ATM) kinase dysfunction, eCIRP-mediated tau phosphorylation, neuroinflammation, metabolic reprogramming, blood–brain barrier disruption, and impaired neurogenesis—through which radiation drives progressive tau pathology and cognitive decline. We discuss shared molecular programs between radiation-exposed and AD brains, the influence of radiation quality, the biphasic hormetic dose–response, and the therapeutic window for low-dose radiotherapy (LDRT) in AD. Finally, we propose that eCIRP inhibition deserves further research as a strategy for both radiation-exposed populations and patients with sporadic AD.

## 2 Epidemiological evidence for radiation–dementia link

A meta-analysis of 18 epidemiological studies spanning five exposure populations found that ionizing radiation significantly increases dementia risk. The pooled analysis yielded a summary relative risk of 1.11 (95% CI: 1.04-1.18; p=0.001) for all-cause dementia at 100-mSv cumulative radiation exposure (10). This estimate encompasses all-cause dementia rather than clinically diagnosed AD specifically, and the included studies varied in diagnostic methods, exposure characterization, and population type, spanning acute medical exposures to chronic environmental exposures. The modest effect size falls within a range where residual confounding cannot be fully excluded. Nonetheless, the consistency of the association across occupational, medical, environmental, and space radiation cohorts supports ionizing radiation as a potentially important environmental dementia risk factor. The following sections examine individual cohorts that contributed to this synthesis (**Figure 1**).

**Figure 1:**
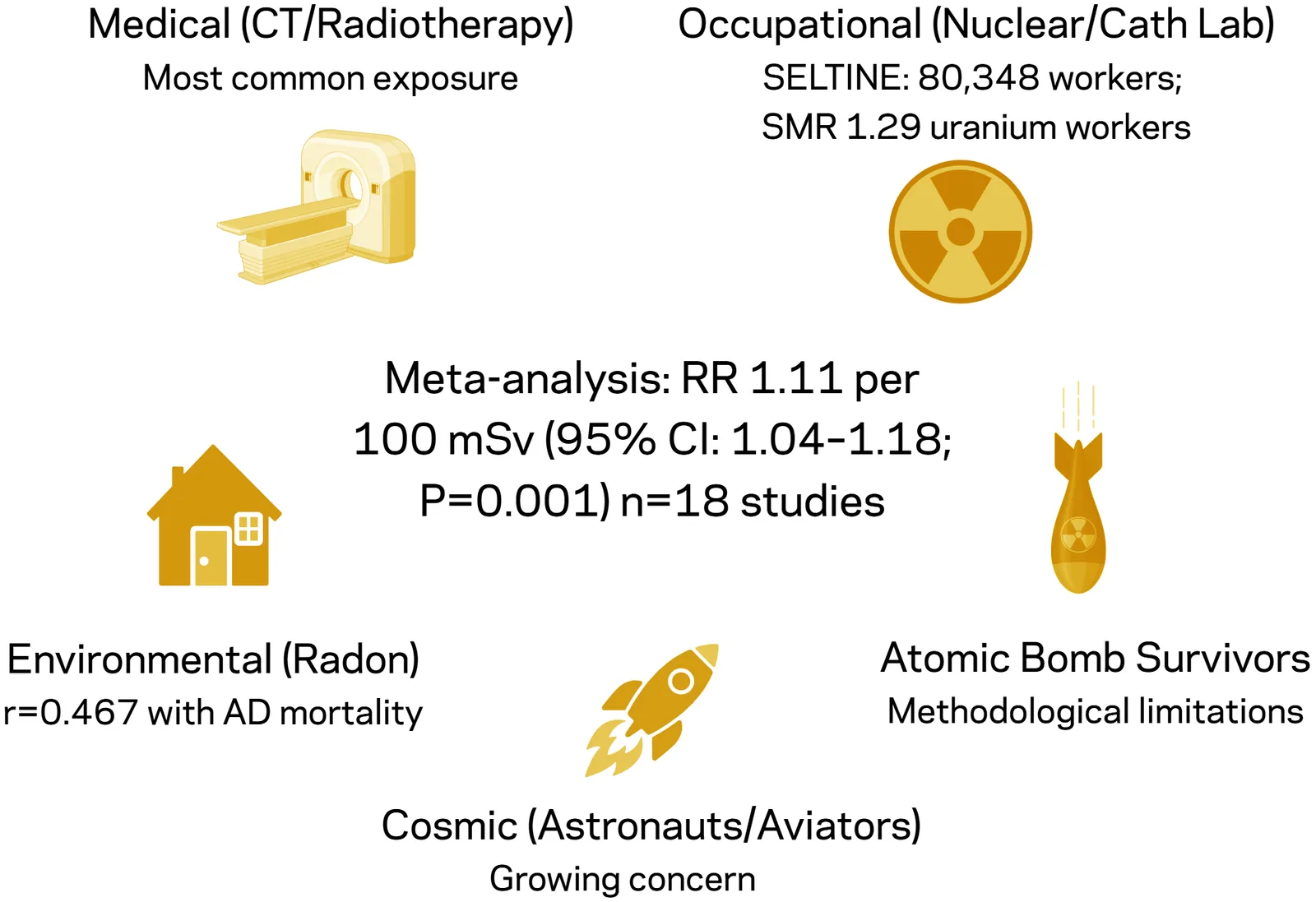
Epidemiological evidence linking ionizing radiation exposure to dementia and Alzheimer’s disease. Occupational cohorts include French nuclear workers (SELTINE; n=80,348), uranium processing facility workers (standardized mortality ratio [SMR] 1.29; 95% CI: 1.04–1.54, among male workers), and cardiac catheterization laboratory personnel (estimated 1–3 Sv lifetime unprotected head dose). Environmental exposure studies demonstrate a statistically significant geographic correlation between residential radon distribution and Alzheimer’s disease (AD) mortality across US states (r=0.467, p=0.001), with autopsy evidence showing selective accumulation of radon decay products in the amygdala (5.98 mSv/year) and hippocampus (1.82 mSv/year). Cosmic radiation exposure in astronauts, airline personnel, and military aviators represents a growing exposure concern. A meta-analysis of 18 independent epidemiological studies across all five exposure populations demonstrated a summary relative risk (RR) of 1.11 (95% CI: 1.04–1.18; p=0.001) for all-cause dementia per 100 mSv cumulative radiation exposure. References (8–10, 13, 14, 16).

### 2.1 Occupational exposure studies

Occupational cohorts are one of five exposure populations in the meta-analysis (10). Several are particularly informative. The SELTINE (Suivi Epidémiologique Longitudinal des Travailleurs de l’Industrie Nucléaire Française) cohort followed 80,348 French nuclear workers from 1968 to 2014 and found a statistically significant association between cumulative occupational ionizing radiation exposure recorded by personal dosimeters and dementia and AD mortality (13). A cohort of over 12,400 uranium processing facility workers in USA and Canada, with employment starting as early as the 1930s and followed until 2017, exhibited elevated incidence of dementia and AD, with a standardized mortality ratio of 1.29 (95% confidence interval: 1.04-1.54) (14). Dose reconstruction for these cohorts relied on facility monitoring records and job-exposure matrices, introducing uncertainties for earlier employment periods (13, 14).

These associations must be interpreted in light of co-exposures inherent to each setting. Nuclear and uranium processing workers encounter heavy metals, organic solvents, and other industrial chemicals that could independently affect cognition (14). Yet, a recent analysis of the Fernald uranium processing cohort specifically evaluated confounding and effect modification by occupational co-exposures and found no evidence that co-exposures confounded the radiation–neurodegenerative mortality association, although machining fluid exposure appeared to modify the radiation effect (15). The healthy worker effect may also influence observed associations. However, the dose–response relationship within the SELTINE cohort links dementia risk specifically to cumulative radiation dose rather than duration of employment or job category (13), and the convergence of associations across diverse occupational settings with different chemical co-exposure profiles (nuclear workers, uranium processors, and catheterization laboratory personnel) supports radiation as the common exposure driving the observed risk. Sex differences in radiation-related dementia risk in occupational cohorts are discussed in Section 5.5.

Personnel working in cardiac catheterization laboratories experience chronic occupational radiation exposure from fluoroscopic procedures with an estimated cumulative unprotected head dose of 1,000 to 3,000 mSv, corresponding to a brain equivalent dose of around 500 mSv (16). It must be noted that these dose estimates carry considerable uncertainty, as they were reconstructed from workload histories rather than direct measurements. A study of 264 catheterization laboratory operators found that 18.2% of exposed individuals showed subnormal olfactory performance, with a notable decline in function correlating with the number of years working in the lab. These data suggest that cumulative radiation exposure causes subtle neurological damage detectable as impaired olfaction (16), which is notable because olfactory decline is an early feature of neurodegenerative diseases including Alzheimer’s (44).

### 2.2 Environmental radiation studies

A geographic analysis of the US population revealed a statistically significant positive correlation between geographic radon distribution and AD mortality rates across different regions (r = 0.467, p = 0.001) (8). As an ecological study, this analysis cannot establish individual-level associations or control for confounders that co-vary geographically with radon levels, such as age distribution, socioeconomic status, or diagnostic practices. The authors hypothesized that inhaled radon could selectively expose the rhinencephalon and hippocampus—structures critical for olfaction and memory—thereby initiating Alzheimer’s pathology. Consistent with this hypothesis, autopsy examination of an 86-year-old woman with AD revealed dramatically elevated concentrations of radon decay products (^210^Po and ^210^Bi) in the amygdala (5.98 mSv/year) and hippocampus (1.82 mSv/year) compared with other brain regions (9). Although this single case cannot be generalized, the selective accumulation in AD-relevant regions rather than diffuse distribution provides a plausible mechanism warranting systematic examination in larger autopsy series. High residential radon exposure is also associated with increased serum levels of 3-methylhistidine, which has been linked to AD (45), and D-sphingosine (46). Together, these observations generate a hypothesis linking environmental radon to AD-relevant brain regions and metabolic pathways, although they fall short of establishing a causal relationship.

### 2.3 Atomic bomb survivor studies and methodological limitations

A longitudinal study of 2,286 atomic bomb survivors from Hiroshima and Nagasaki with biennial medical examinations found no significant relationship between radiation dose and dementia incidence, including AD and vascular dementia, even at doses up to 4 Gy (47, 48). However, the original Life Span Study dose estimates significantly underestimated true exposure by neglecting residual radiation from radioactive black rain fallout (49). A recent analysis of 43,056 Hiroshima survivors confirmed that residual radiation made a substantial, previously unaccounted contribution to health outcomes, with estimated residual doses in downwind districts exceeding initial dose estimates (50). This dose misclassification would have contaminated the reference group with radiation-exposed individuals, biasing results toward the null. Additional factors that may have attenuated a true association include the healthy survivor effect, limited diagnostic sensitivity for dementia in the early follow-up decades, and the extended latency between radiation exposure and clinical dementia. At the same time, many survivors received substantial doses, and a strong radiation-dementia effect would likely have been detected despite these limitations. Despite the null finding, Srivastava’s meta-analysis detected increased dementia risk even when the atomic bomb survivor data are included (10), indicating that the overall weight of evidence supports a radiation–dementia association.

## 3 Mechanistic evidence: how radiation may cause dementia

Having discussed epidemiological evidence, we now turn to the molecular mechanisms by which radiation may drive neurodegeneration, focusing on eCIRP-mediated neuroinflammation. Notably, eCIRP is elevated in the cerebrospinal fluid (2.6-fold) and plasma of both AD patients and irradiated animals (35, 41), pointing to a shared pathway between radiation injury and AD. The proposed relationship between radiation-induced DNA damage, ROS generation, microglial activation, and eCIRP release is depicted in **Figure 2A**.

**Figure 2:**
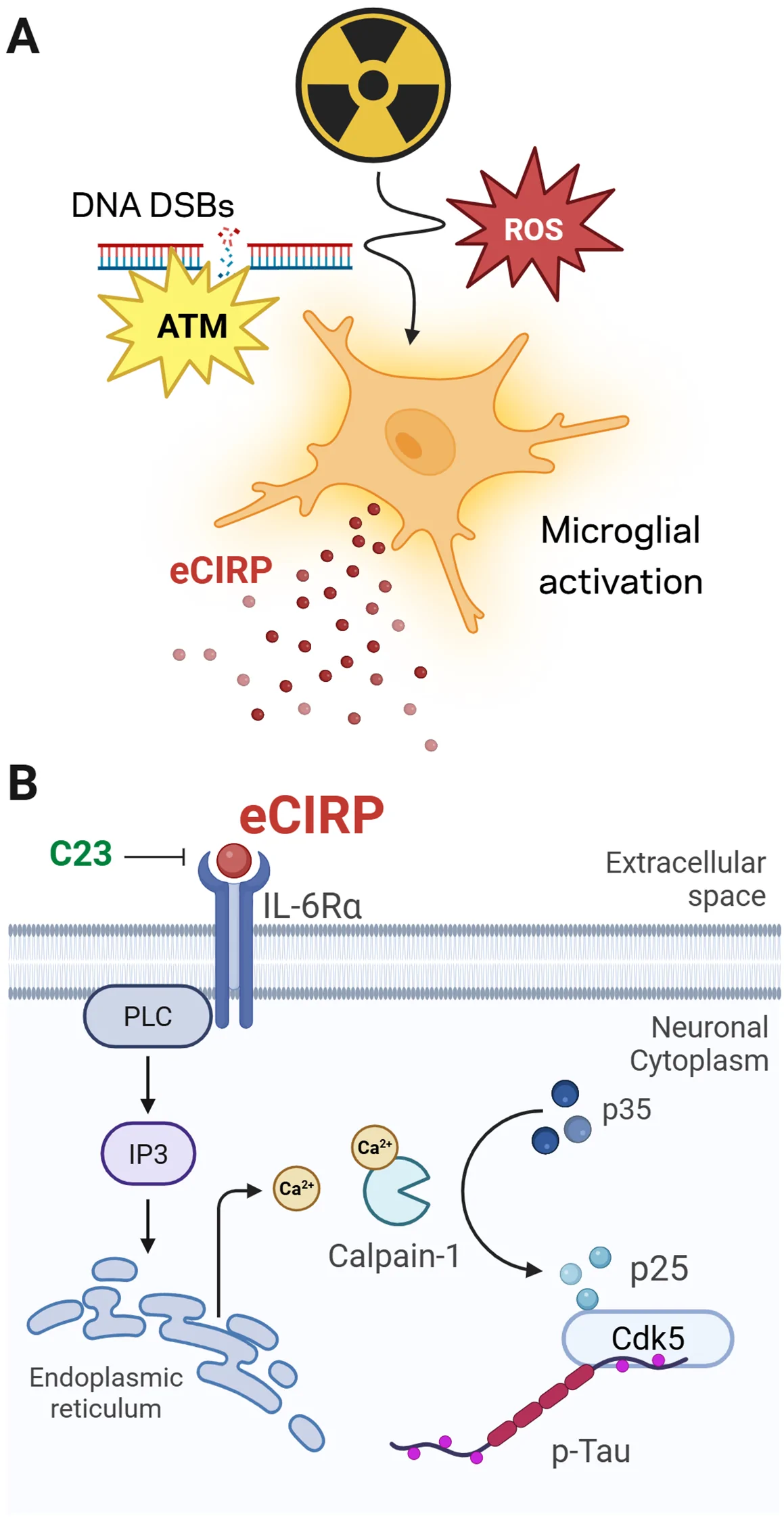
Radiation-induced eCIRP release and the neuronal tau phosphorylation cascade. **(A)** Ionizing radiation generates DNA double-strand breaks (DSBs) and reactive oxygen species (ROS) that activate ataxia-telangiectasia mutated (ATM) kinase and trigger microglial activation. ROS and ATM activation are proposed to trigger nuclear-to-cytoplasmic translocation and extracellular release of cold-inducible RNA-binding protein (eCIRP). Quantitative data: eCIRP increases 2.6-fold in CSF, 1.2-fold in serum, and hippocampal CIRP protein increases 65% at 48 h post-irradiation (p ≤ 0.05). See Section 2.1–2.3 for mechanistic details. References (35, 40–42). **(B)** Released eCIRP binds neuronal IL-6Rα (inhibited by C23), activating PLC → IP3 → Ca²^+^ release from endoplasmic reticulum → calpain-1-mediated cleavage of p35 to p25 → Cdk5 hyperactivation → tau phosphorylation at Ser199, Ser202, and Ser396. Validated by CIRP-knockout mice, intracerebroventricular eCIRP injection, and C23 inhibitor studies. See Section 2.2 for full mechanistic description. References (35, 40–42).

### 3.1 Oxidative stress, DNA damage, and the ATM pathway

Exposure to ionizing radiation generates reactive oxygen species (ROS) that directly oxidize and damage lipid membranes, proteins, and DNA; when antioxidant defenses are overwhelmed, cellular dysfunction ensues. Since oxidative stress is also central to AD pathogenesis (51), radiation-induced ROS production may initiate or accelerate the same neurodegenerative cascade.

Radiation-induced DNA double-strand breaks activate the ataxia-telangiectasia mutated (ATM) protein kinase. Activated ATM phosphorylates CREB on a stress-inducible domain, impairing CREB’s ability to activate genes required for cell survival and homeostasis. Since CREB governs genes involved in neuronal survival and homeostasis, radiation-induced disruption of ATM-CREB signaling could contribute to the neuronal loss seen in neurodegeneration (52). ATM protein kinase also becomes progressively impaired with age and has been implicated in AD pathogenesis (53). Reduced neuronal ATM signaling in AD brain regions such as hippocampus and frontal cortex results in aberrant cell cycle reentry and apoptotic death (54). Impaired ATM activity also drives increased amyloid precursor protein (APP) expression and synaptic dysfunction, intersecting with neuroinflammatory pathways that compound neuronal damage (55–59).

In the developing central nervous system, ionizing radiation induces widespread apoptotic cell death in diverse brain regions including the cerebellum, which is mediated by a requirement for the ATM protein kinase, since *Atm*-null mice show remarkable resistance to radiation-induced apoptosis in the developing nervous system (60). The same ATM-dependent pathway could, in principle, be engaged by the lower chronic exposures encountered in occupational or environmental settings. In *Atm*-null mice, tissues that develop pathological changes accumulate substantial oxidative damage, with cerebellar Purkinje cells showing particularly severe oxidative modifications despite the absence of mutations in genes directly involved in oxidative defense (61). Loss of ATM function therefore permits oxidative damage to accumulate in vulnerable neuronal populations, and individuals with reduced ATM activity may be predisposed to radiation-induced dementia as a result. In human neural stem cells, ATM depletion by shRNA reduced oligodendrocyte generation and increased susceptibility of the remaining oligodendrocytes to oxidative stress, whereas neuronal differentiation was unaffected (62). This cell-type-specific vulnerability suggests that ATM dysfunction may preferentially damage the myelinating cells needed for white matter integrity, consistent with the demyelination observed in radiation-induced cognitive impairment.

### 3.2 Tau phosphorylation and amyloid pathology

Exposure of primary cortical neurons to ionizing radiation (0.5 or 2 Gy gamma rays) caused dose- and time-dependent increases in tau protein phosphorylation at multiple phosphorylation sites, accompanied by activation of GSK3β, c-Jun N-terminal kinase (JNK), and extracellular signal-regulated kinase (ERK), three kinases known to phosphorylate tau in AD (63). These results establish that ionizing radiation can reproduce the pathological tau modifications found in AD brain. A molecular investigation using Western blotting and digital PCR in rhesus macaque brains up to 60 days following a single 5.8-Gy total-body gamma radiation exposure detected significantly lower levels of multiple soluble phosphorylated-tau species (pTau^181^ and pTau^217^) in the frontal cortex, hippocampus, and cerebellum of irradiated animals compared with controls (64). While this reduction in phosphorylated tau seems counterintuitive, it was accompanied by increased neurofilament-light chain levels and decreased myelin basic protein expression, suggesting that acute radiation exposure triggers complex molecular rearrangements in tau phosphorylation dynamics and neuronal structural protein expression that precede overt neurodegeneration (64). PET imaging data offer complementary evidence for radiation-induced microtubule disruption in non-human primates. Dual-tracer PET using [¹¹C]PiB (amyloid) and [¹¹C]MPC-6827 (microtubule stability) in seven irradiated and seven control rhesus macaques revealed significantly increased [¹¹C]MPC-6827 uptake in the occipital lobe of irradiated animals (p < 0.0001), indicating region-specific microtubule destabilization, whereas no significant differences in amyloid deposition were detected (65). This dissociation between microtubule injury and amyloid pathology is consistent with the amyloid-independent tau phosphorylation driven by the eCIRP pathway.

In contrast to this later-phase reduction, mice exposed to 10-Gy total-body X-rays developed rapid AD-like tau phosphorylation, with phosphorylated tau increasing 2.5-fold at 48 h and remaining elevated through day 7 (35). Radiation increased eCIRP levels 2.6-fold in cerebrospinal fluid and 1.2-fold in serum (35). The released eCIRP bound neuronal IL-6Rα—a well-established eCIRP receptor (66)—and triggered phospholipase C (PLC) activation and inositol 1,4,5-trisphosphate (IP3)-dependent calcium release from endoplasmic reticulum stores (40). The resulting calcium mobilization activated calpain-1, which cleaved the Cdk5 regulatory subunit p35 into its truncated, hyperactivating form p25. The p25/Cdk5 complex then phosphorylated tau at pathological sites Ser199, Ser202, and Ser396—sites identical to those found in AD neurofibrillary tangles (35). Loss-of-function studies in CIRP-knockout mice showed marked reduction in p25 and phosphorylated tau, whereas treatment with the eCIRP inhibitor C23 abrogated radiation-induced tau phosphorylation, suggesting a therapeutic approach with potential to mitigate radiation-induced dementia (35). Whether eCIRP-mediated tau phosphorylation leads to cognitive impairment in irradiated animals has not yet been directly tested. However, eCIRP has been shown to mediate brain hypoactivity and impaired cognition in alcohol exposure models (39), and the tau phosphorylation sites induced by eCIRP (Ser199, Ser202, and Ser396) are the same sites whose phosphorylation correlates with cognitive decline and neurofibrillary tangle formation in AD patients (23–26, 28). Therefore, radiation-induced eCIRP release drives pathological tau phosphorylation (**Figure 2B**), which can then be modulated by phosphatase activity, autophagy, or aggregation into insoluble tangles.

The apparent contradiction between reduced phosphorylated tau in irradiated non-human primates and increased phosphorylated tau in irradiated mice reflects different measurement timepoints in a biphasic radiation response: Chaung et al. captured acute kinase-driven phosphorylation at 48 h post-irradiation when eCIRP and Cdk5 peak (35), whereas Murphy et al. measured tau up to 60 days later (with some animals euthanized earlier due to radiation-induced moribundity) when phosphorylated tau may have undergone dephosphorylation, clearance, or aggregation into insoluble forms (64). In addition, methodological differences in tau extraction protocols, species differences between primates and rodents, and systemic complications in the primate study further explain the divergence, with both studies demonstrating that ionizing radiation fundamentally alters tau metabolism through temporally distinct pathways relevant to AD. This reconciliation remains inferential; the contradictory directional effects have not been experimentally resolved.

Exposure of 7-week-old transgenic mice overexpressing human amyloid precursor protein (APP23 mice) to brain-only ^56^Fe particle radiation (600 MeV/nucleon at doses of 1, 2, or 4 Gy) significantly accelerated the onset of age-related electrophysiological abnormalities in hippocampal synaptic transmission, advancing the appearance of deficits in synaptic plasticity from 14 months of age in sham-irradiated mice to 9 months of age in irradiated mice (67). High-energy particle radiation thus accelerates the onset of Alzheimer’s-related neurological deficits in this amyloid-overexpressing model. The temporal dynamics of eCIRP-mediated tau phosphorylation differ from the canonical amyloid cascade sequence in sporadic AD, where amyloid-β accumulation precedes tau pathology. Radiation-induced eCIRP drives tau phosphorylation within hours (35), well before amyloid plaque formation could occur, suggesting that the eCIRP pathway can drive tau pathology through an amyloid-independent mechanism. This matters clinically, as current anti-amyloid therapies (lecanemab and donanemab) show that modest efficacy and amyloid-independent mechanisms of tau pathology remain a major therapeutic frontier. At the same time, eCIRP and amyloid-β pathology likely intersect at multiple levels: ATM dysfunction drives APP accumulation (55, 58, 59), amyloid-β itself induces eCIRP release from glia (40), and radiation accelerates electrophysiological deficits in amyloid-overexpressing mice (67). Whether eCIRP inhibition can reduce tau pathology in the setting of ongoing amyloid accumulation and whether eCIRP-targeted therapy would complement anti-amyloid approaches are open questions with important therapeutic implications.

### 3.3 Neuroinflammation and metabolic reprogramming

In addition to driving tau phosphorylation through the IL-6Rα/Cdk5 cascade described in Section 2.2, eCIRP likely promotes radiation-induced cognitive impairment through multiple additional mechanisms. eCIRP activates toll-like receptor 4 (TLR4) and its essential co-receptor myeloid differentiation factor 2 (MD2) complex as well as triggering receptor expressed on myeloid cells 1 (TREM1) in peripheral immune cells (36), and these same receptors are expressed on microglia and other brain-resident cells (68–70), potentially positioning eCIRP as a driver of neuroinflammatory amplification through M1 microglial polarization (71), neurotoxic A1 astrocyte induction (40), endothelial cell activation and programmed cell death (72–74), blood–brain barrier (BBB) disruption (74), suppression of hippocampal neurogenesis (discussed in Section 2.5) (75), synaptic dysfunction (76, 77), and impaired amyloid-β clearance (78). The known biology of eCIRP as a multireceptor DAMP (36), together with the expression of these receptors in the CNS, provides strong mechanistic rationale for these additional pathways, although each remains to be demonstrated directly in the irradiated brain. Since eCIRP activates the same immune cells that produce it (36, 79), a positive feedback loop likely develops in which radiation-initiated eCIRP release perpetuates neuroinflammation well after the initial insult has passed.

Ionizing radiation also reprograms microglial metabolism, shifting energy production from oxidative phosphorylation to aerobic glycolysis. This metabolic switch increases lactate output, dysregulates lipid droplet accumulation, and amplifies neuroinflammatory signaling (80). The resulting chronic neuroinflammation impairs neurogenesis and oligodendrocyte generation, linking radiation-induced microglial dysfunction to cognitive decline. eCIRP appears to be both a product and a driver of this metabolic reprogramming. In a trauma model, eCIRP activates NADPH oxidase through TLR4-MyD88 signaling in macrophages, generating ROS that activate endonuclease G and cause mitochondrial DNA (mtDNA) fragmentation (81). The fragmented mtDNA then triggers both p62-related autophagy and necroptosis, with autophagy serving as a counterbalancing mechanism that suppresses necroptotic cell death and pro-inflammatory responses (81). This axis directly links eCIRP to mitochondrial damage in myeloid cells, with clear relevance to brain microglia, which express the same TLR4/MyD88 machinery. In the brain, mtDNA fragmentation could activate the cGAS-STING pathway, amplifying type I interferon responses and sustaining neuroinflammation.

Building on this connection between eCIRP and mitochondrial injury, radiation-induced eCIRP directly impairs mitochondrial oxidative phosphorylation in macrophages, causing marked reductions in basal and maximal respiration and ATP production that drive ferroptosis—characterized by elevated lipid peroxidation and downregulation of glutathione peroxidase 4 (GPX4) (82). These effects were time-dependent, correlating with progressive eCIRP accumulation at days 3 and 5 post-irradiation, and were recapitulated by direct eCIRP treatment alone, establishing that eCIRP alone is sufficient to cause mitochondrial dysfunction (82). The novel opsonic eCIRP inhibitor MOP3 restored mitochondrial function, reduced ferroptosis, and improved phagocytic capacity in irradiated macrophages (82), paralleling the protective effects of C23 on tau phosphorylation and further supporting eCIRP as a druggable target. Together, the Li et al. and Zhou et al. studies delineate two temporally and mechanistically distinct phases of eCIRP-mediated mitochondrial injury: an early phase involving TLR4-dependent ROS generation and mtDNA fragmentation (81), and a later phase involving progressive respiratory chain failure, ferroptotic lipid peroxidation, and functional impairment (82).

eCIRP-mediated mitochondrial dysfunction is not confined to macrophages or to the radiation context. In hepatic ischemia–reperfusion injury, CIRP promotes oxidative stress and mitochondrial fission (83), indicating that eCIRP-driven metabolic impairment is conserved across organ systems and stressors. Furthermore, lactate accumulation during inflammatory states promotes lactylation of intracellular CIRP in macrophages, facilitating its extracellular release—a mechanism demonstrated in sepsis-induced acute lung injury where the lactate–CIRP–ZBP1 axis drives pulmonary endothelial cell PANoptosis (84). In the radiation context, this finding suggests that the glycolytic shift and resultant lactate production in irradiated microglia may amplify eCIRP release through posttranslational lactylation, creating a metabolic feedforward loop. This self-reinforcing cycle could perpetuate neuroinflammation well beyond the initial radiation exposure.

Whether these mechanisms operate in brain-resident microglia as they do in peripheral macrophages remains to be tested directly. However, microglia share the same myeloid lineage, express eCIRP receptors TLR4/MyD88, TREM1, and IL-6Rα, and undergo similar metabolic shifts during neuroinflammation. If the eCIRP–TLR4–mtDNA fragmentation axis (81) and the eCIRP–mitochondrial dysfunction–ferroptosis axis (82) are operative in irradiated microglia, they would provide additional mechanisms by which eCIRP perpetuates the chronic neuroinflammatory state that drives tau pathology, disrupts the BBB, suppresses neurogenesis, and impairs amyloid-β clearance in the radiation-exposed brain.

### 3.4 Blood–brain barrier disruption

Radiation damages the blood–brain barrier through multiple mechanisms, including endothelial cell apoptosis, oxidative stress, neuroinflammation, endothelial cell dysfunction, arterial stiffening, atherosclerosis, and cerebral amyloid angiopathy (85). These vascular injuries impair nutrient delivery and toxic metabolite clearance, increasing the risk of cerebrovascular disease and consequent dementia through mechanisms independent of or synergistic with direct neuronal damage. Cerebrovascular changes induced by cosmic radiation closely parallel the vascular dysfunction observed in AD (86).

Recent work positions eCIRP as a direct mediator of endothelial injury, potentially connecting radiation-induced neuroinflammation to BBB breakdown. Intravenous recombinant CIRP causes TREM1-dependent endothelial activation—upregulating ICAM-1, increasing cytokine production, and releasing soluble TREM1 (87). TREM1-knockout mice are protected, and the TREM1 inhibitory peptide M3 attenuates the response *in vitro* (87). Circulating eCIRP can therefore directly activate vascular endothelium through TREM1, a mechanism that likely extends to the cerebral vasculature, where TREM1 is also expressed (74). eCIRP also causes direct TLR4-dependent endothelial injury. In HUVECs, recombinant eCIRP reduced NO production and eNOS activity, increased ROS and lipid peroxidation, upregulated inflammatory cytokines, and promoted monocyte adhesion in a TLR4-dependent manner (88). eCIRP was also elevated in high-fat-diet-fed ApoE^-/-^ mice and correlated with atherosclerotic plaque burden, and serum CIRP was higher in patients with coronary heart disease (88). These findings establish that eCIRP can directly impair endothelial NO bioavailability and redox homeostasis through TLR4, providing a vascular injury mechanism distinct from the neutrophil-mediated PANoptosis pathway described below.

eCIRP also amplifies vascular injury indirectly through its effects on neutrophils. In sepsis-induced acute lung injury, eCIRP induces a DLL4-expressing neutrophil population that triggers endothelial PANoptosis—simultaneous activation of pyroptosis, apoptosis, and necroptosis—through Notch1-ZBP1 signaling in pulmonary vascular endothelial cells (72). DLL4+ neutrophils upregulate ZBP1, cleaved gasdermin D, cleaved caspase-3, and phosphorylated MLKL in endothelial cells, exacerbating vascular barrier dysfunction. Indeed, a novel Notch1-DLL4 inhibitor (NDI) reduced endothelial PANoptosis, decreased vascular permeability, and improved survival (72). These eCIRP-induced DLL4+ neutrophils also cause ADAM-17-mediated endothelial barrier disruption (73). While these pathways were characterized in pulmonary vasculature, they have direct implications for cerebrovascular injury after radiation: radiation mobilizes neutrophils and elevates circulating eCIRP (35), and the cerebral microvasculature is particularly vulnerable to inflammatory insult. Endothelial TREM1 activation has been directly shown to mediate BBB disruption and cognitive impairment in sepsis through the PI3K/Akt pathway (74), providing precedent for eCIRP–TREM1-mediated BBB compromise.

Together, these studies show that eCIRP damages the vasculature through at least two complementary mechanisms: direct TREM1-mediated endothelial activation (87) and indirect, neutrophil-mediated endothelial PANoptosis (72). In the brain, where BBB integrity is essential for maintaining the neural microenvironment and clearing amyloid-β, these eCIRP-driven vascular injuries could synergize with the neuronal tau phosphorylation pathway (Section 2.2) and the neuroinflammatory and metabolic reprogramming described above (Section 2.3), such that neurons, microglia, and endothelial cells are all damaged by the same radiation-induced DAMP.

### 3.5 Neurogenesis impairment

Impaired hippocampal neurogenesis is a shared feature of AD, Parkinson’s disease, and age-related cognitive decline. Proinflammatory cytokines produced by activated microglia—including interleukin-6, tumor necrosis factor-α, reactive oxygen species, and nitric oxide—directly suppress neurogenesis (89). Since radiation activates microglia and increases ROS production, radiation-induced inflammation may suppress adult hippocampal neurogenesis—yet another route to cognitive decline. The mechanism of neurogenesis impairment appears to involve cell cycle arrest rather than stem cell death: subventricular zone neural stem cells maintained self-renewal after 8-Gy γ-irradiation in both culture and *in vivo*, but the proliferation of their multipotent progeny was markedly reduced (90). Radiation thus impairs neurogenesis by slowing the expansion of transit-amplifying progenitors rather than by eliminating the stem cell pool itself. A histopathological analysis of rat brain tissue 18–21 weeks after fractionated whole-brain irradiation (35 Gy in seven 5-Gy fractions) revealed marked inhibition of neurogenesis in the hippocampal dentate gyrus and subventricular zone–olfactory bulb axis, accompanied by evidence of increased neurodegeneration, strong astrogliosis, and metabolic changes including decreased N-acetylaspartate and γ-aminobutyric acid levels detected by proton magnetic resonance spectroscopy (91). These findings provide direct evidence that radiation causes lasting damage to neurogenesis and neuronal viability in brain regions critical for learning and memory.

### 3.6 Shared molecular pathways in radiation and AD

The mechanistic pathways described above share notable molecular features with AD. A microarray analysis identified nine neural signaling pathways with high transcriptional response concordance between mouse brain collected within hours of low-dose radiation exposure, unirradiated aging human brain, and unirradiated brain tissue from AD patients (92). These shared pathways included regulation of ion channels, long-term potentiation and depression (LTP and LTD, respectively), and vascular dysfunction (92). This study suggests that ionizing radiation induces the same pathological gene expression patterns that emerge during normal aging and AD development (92). An integrated metabolomic and transcriptomic analysis of mouse hippocampus 14 days after 0.5-Gy X-ray exposure identified significant alterations in 15 metabolites, with tyrosine metabolism emerging as the most perturbed pathway through correlated gene–metabolite interactions; these metabolomic changes overlapped with pathways implicated in Alzheimer’s and Parkinson’s disease pathogenesis (93). These metabolic disruptions at low doses may contribute to the shared molecular programs between radiation injury and neurodegeneration.

The eCIRP/IL-6Rα/Cdk5 cascade described in Section 2.2 may serve as a mechanistic nexus connecting these shared transcriptional responses, with eCIRP-driven neuroinflammation acting as a convergent pathway through which both radiation and genetic risk factors promote tau pathology (35, 40–42). At the epigenetic level, miR-132 and miR-29—microRNAs that regulate common downstream pathways including neurogenesis and learning and memory—are altered after ionizing radiation exposure and are also dysregulated in AD, depression, and schizophrenia (94). Taken together, these studies support the existence of shared molecular programs between radiation-induced brain dysfunction and neurodegeneration.

### 3.7 Radiation-induced cognitive impairment in clinical populations

The mechanistic pathways described above operate within a well-established clinical context: radiation-induced cognitive impairment affects 50%–90% of long-term brain tumor survivors and is recognized as a significant clinical consequence of cranial radiotherapy (85). Established mechanisms include white matter demyelination, microvascular rarefaction, hippocampal neuronal loss, and neurotransmitter alterations (85, 91). Clinical interventions including memantine and hippocampal-avoidance radiotherapy provide partial neuroprotection, demonstrating that radiation-induced cognitive decline is mechanistically addressable. eCIRP has not been examined in these clinical populations but could represent a complementary therapeutic target. Measuring eCIRP levels in cerebrospinal fluid of patients undergoing cranial radiotherapy would test the clinical relevance of this pathway and could identify patients who might benefit from eCIRP-targeted neuroprotection.

### 3.8 Dose considerations in mechanistic studies

The eCIRP/IL-6Rα/Cdk5 pathway was characterized at 10 Gy (35), whereas other key studies used 0.5–2 Gy (63), 5.8 Gy (64), or 17 Gy (Section 4.1) (95). The absorbed doses of 0.5–10 Gy delivered acutely in these studies are orders of magnitude higher than the cumulative effective doses of ~100 mSv (approximately 0.1 Gy for low-LET radiation) associated with the increase in dementia risk in Srivastava’s meta-analysis (10). These doses are typical of initial mechanistic and proof-of-concept investigations, which require robust, measurable biological responses for pathway identification. However, the uncertainty inherent in extrapolating from acute high-dose experimental exposures to chronic LDIR human exposures must be acknowledged. Biological responses at high doses may not scale linearly to low doses, particularly given the involvement of dose-rate-dependent repair mechanisms, adaptive responses, and potential threshold effects. Although radiation-induced tau phosphorylation has been demonstrated at doses as low as 0.5 Gy in primary neurons (63), the minimum dose at which eCIRP is released and the shape of the dose–response curve for eCIRP-mediated neuroinflammation remain unknown. Establishing dose–response relationships for eCIRP release across the 0.01–1-Gy range—spanning occupationally and environmentally relevant exposures—is a critical priority for translating these mechanistic findings to human risk assessment.

In addition to absorbed dose, the rate and geometry of dose delivery influence the biological response and further complicate extrapolation from experimental to human settings. The mechanistic studies cited herein employed acute, high-dose-rate exposures (e.g., 1 Gy/min for the eCIRP study (35)), whereas occupational and environmental exposures are typically chronic and delivered at dose rates many orders of magnitude lower (e.g., <0.001 mGy/h for background radiation). Low-dose-rate exposures permit concurrent DNA repair between ionization events and may not generate the burst of ROS and eCIRP release observed after acute irradiation. Similarly, whole-body versus localized (e.g., cranial-only) irradiation fields produce different systemic responses, including differences in immune cell mobilization and circulating DAMP levels. These variables complicate direct comparisons between experimental and epidemiological data and underscore the need for mechanistic studies employing chronic, low-dose-rate protocols that more faithfully recapitulate human exposure scenarios.

### 3.9 Radiation quality considerations

The studies reviewed here span a wide range of radiation qualities: the eCIRP/tau phosphorylation pathway was characterized using low-LET X-rays (250 kVp) (35); tau kinase activation was demonstrated with γ-rays (^60^Co) (63, 64); hippocampal electrophysiological deficits were induced by high-LET ^56^Fe ions (600 MeV/nucleon) (67); and the epidemiological radon associations involve alpha particles from ²²²Rn decay products (8, 9). These radiation types differ substantially in LET—from ~2 keV/μm for X-rays and γ-rays to ~150 keV/μm for ^56^Fe ions and ~100–200 keV/μm for alpha particles—resulting in qualitatively different patterns of DNA damage (sparse vs. clustered), different efficiencies of ROS generation, and different relative biological effectiveness (RBE) values that are endpoint-dependent. Consequently, the biological responses observed with one radiation type cannot be directly extrapolated to another without accounting for these differences. Whether eCIRP release is differentially triggered by high- vs. low-LET radiation has not been examined and represents an important gap, particularly for risk assessment in populations exposed to mixed-field radiation environments such as cosmic radiation during spaceflight.

## 4 Dose–response relationship: the hormesis paradox

The dose–response relationship between ionizing radiation and brain pathology presents an apparent paradox: the same physical agent that causes dementia-like neurodegeneration at high doses may exert neuroprotective effects at low doses, and LDRT has been proposed as a therapeutic intervention in AD. Reconciling these observations requires distinguishing between two fundamentally different biological contexts—the “double-edged sword” of radiation, which appears detrimental to the healthy brain but potentially therapeutic to the diseased brain (96). In the causation context, ionizing radiation acts as an environmental stressor that generates DNA double-strand breaks, oxidative stress, and eCIRP-mediated neuroinflammation, driving pathological tau phosphorylation and progressive neurodegeneration through the mechanisms described in Section 2. In the therapeutic context, controlled LDRT is deliberately administered to an already-diseased brain to activate endogenous neuroprotective pathways that suppress the existing neuroinflammatory milieu of AD. The two contexts differ in dose, in the baseline state of the brain at the time of exposure, in the direction of the dominant biological response, and in whether the outcome is protective or pathological. The following sections examine the biphasic dose–response relationship and its mechanistic basis, with the distinction between radiation as cause and radiation as treatment explicitly maintained throughout.

### 4.1 Biphasic dose–response (hormesis)

Hormesis is a biological phenomenon in which low doses of a stressor elicit beneficial adaptive responses whereas higher doses cause harm, producing a non-linear biphasic dose–response relationship. In the context of ionizing radiation and brain health, this pattern manifests as a dose-dependent divergence in molecular outcomes: low doses activate neuroprotective signaling cascades, whereas higher doses overwhelm cellular defenses and drive neurodegeneration through the eCIRP-mediated and oxidative stress pathways described in Section 2. A study examining the effects of continuous low-dose X-ray exposure on the developing brain of young rats found that exposure to 0.2 mGy/day for 7 days significantly increased the expression of microtubule-associated protein 2 (MAP-2) and promoted the development of microtubules and normal synaptic structures in hippocampal CA1 neurons, whereas exposure to 1.0 mGy/day resulted in neuronal degeneration, reduced MAP-2 expression, destruction of microtubules, and damage to postsynaptic density (97). Proteomic analysis of mouse hippocampus tissue collected 24 months after total-body irradiation with doses of 0.063, 0.125, or 0.5 Gy found that the two lower doses activated the cAMP response element binding protein (CREB) signaling pathway and reduced the number of activated microglia while decreasing proinflammatory markers, whereas the higher dose deactivated CREB signaling and induced prominent microglia and astrocyte activation, neuroinflammation, and apoptotic markers (98). Since CREB signaling plays a central role in learning and memory, these findings suggest that the threshold between neuroprotective and neurodegenerative radiation responses is determined by the balance between protective CREB activation at low doses and pathological eCIRP release and neuroinflammation at higher doses—two mechanistically distinct pathways that diverge at intermediate dose levels. Notably, chronic low-dose-rate radiation in an Alzheimer’s-susceptible background also alters CREB signaling. ApoE-deficient mice exposed to 1 or 20 mGy/day for 300 days (cumulative doses 0.3 and 6.0 Gy) showed hippocampal phosphoproteome changes affecting synaptic plasticity, calcium signaling, and brain metabolism, with CREB signaling inhibited at both dose rates (99). Thus, chronic occupationally representative exposures can engage the same molecular targets as acute irradiation in a genetically susceptible background. This dose–response pattern applies to radiation acting on a healthy brain and should not be conflated with the therapeutic use of LDRT in the already-diseased Alzheimer’s brain (Section 3.3). The available data suggest a tentative dose–response framework: doses at or below approximately 0.125 Gy activate CREB signaling and suppress microglial activation (98), doses of 0.5 Gy and above activate tau kinases (63), and 10 Gy triggers robust eCIRP release and the full IL-6Rα/Cdk5 cascade (35). Characterizing eCIRP release across the 0.1–2-Gy dose range would define where the switch from neuroprotective to neurotoxic radiation responses occurs at the molecular level and clarify the relevance of the eCIRP pathway to occupational and environmental exposures.

### 4.2 Fractionation advantage

The temporal pattern of radiation delivery significantly influences the brain’s capacity to mount protective responses and activate DNA repair mechanisms, independent of total dose. A comparative study examining the effects of fractionated versus acute whole-body gamma irradiation on rat brain found that acute doses of 6 or 8 Gy caused greater DNA damage, more extensive neurodegeneration markers, and more pronounced downregulation of neuroprotective cytokines compared with the same total dose delivered in four fractions over 4 days (100). This fractionation advantage is mechanistically distinct in the causation and treatment contexts: in the causation context, fractionated environmental or occupational exposure allows partial DNA repair and antioxidant recovery between exposures, reducing cumulative eCIRP release and neuroinflammatory burden; in the treatment context, fractionated delivery of LDRT is designed to repeatedly activate neuroprotective CREB signaling and anti-inflammatory microglial polarization while remaining below the threshold that triggers pathological eCIRP release. In both contexts, what matters is whether a given fraction activates protective or pathological pathways, making dose rate and fractionation as important as total dose in determining outcome.

### 4.3 Therapeutic window: LDRT as treatment for AD

The potential therapeutic application of LDRT in AD is conceptually distinct from the causation evidence discussed above. LDRT is administered intentionally to an already-diseased brain to exploit endogenous neuroprotective mechanisms that are specifically deficient in AD and different from those involved in dementia initiation. The Alzheimer’s brain has a pathological baseline—chronic neuroinflammation, impaired CREB signaling, microglial dysfunction—in which LDRT may restore protective signaling rather than trigger damage.

A study of ionizing radiation effects (0.1 Gy × 10 fractions, total 1 Gy) in 5xFAD transgenic mice (a model of AD with amyloid-β overexpression) found that radiation exposure promoted the polarization of microglia from the proinflammatory M1 phenotype toward the anti-inflammatory M2 phenotype through activation of the triggering receptor expressed on myeloid cells 2 (TREM2), which was accompanied by reduced levels of proinflammatory cytokines (tumor necrosis factor-α, interleukin-1β, and interleukin-6) and reduced amyloid-β deposition (101). The shift toward anti-inflammatory microglial activation suggests one mechanism by which controlled radiation might reduce neuroinflammation in AD, although clinical relevance remains to be established. A mechanistic study of low-moderate dose ionizing radiation (total 9 Gy in five 1.8-Gy fractions) in 5xFAD transgenic mice found that radiation did not reduce amyloid-β accumulation at 4 days postexposure but significantly reduced synaptic degeneration and neuronal loss in the hippocampus and cerebral cortex and reduced nuclear factor kappa-light-chain-enhancer of activated B cell (NF-κB) pathway activation in microglial cells (102). In another transgenic model, a single 4-Gy gamma dose in APP/PS1 mice improved behavioral impairment, reduced levels of APP, phosphorylated tau, and BACE, decreased neurofibrillary tangle formation, and increased antioxidant enzyme expression, with the effects attributed to potentiation of insulin signaling through GSK3β(Ser9) upregulation and consequent suppression of ERK and JNK phosphorylation (103). These findings extend the therapeutic window to a moderate single dose and implicate insulin signaling as a pathway for radiation-mediated tau dephosphorylation. In a tauopathy model, combined ionizing radiation (0.24-Gy γ-rays plus 0.18-Gy ¹²C ions) improved locomotor abilities in Tau P301S mice and modulated both pro- and anti-inflammatory cytokines in the cerebellum and hippocampus (104). These data suggest that therapeutic radiation effects extend beyond amyloid-driven models to tau-driven neurodegeneration as well.

These preclinical observations are consistent with a hormetic dose–response in AD models, but important caveats apply. Radiation hormesis has not been validated for neurodegenerative endpoints in humans, and the possibility that LDRT simultaneously activates protective pathways while initiating subclinical damage that manifests only after long latency cannot be excluded. Notably, the preclinical doses that produced neuroprotective effects in AD models (0.1–9 Gy total) overlap with dose ranges associated with cognitive impairment in cranial radiotherapy survivors (85). Therapeutic claims should not be extrapolated beyond the specific experimental conditions tested.

An uncontrolled pilot study of LDRT in a small number of Alzheimer’s patients reported qualitative cognitive improvement (105), providing preliminary feasibility and a rationale for controlled trials with validated outcome measures. A separate case report described an early-stage Alzheimer’s patient who exhibited temporary improvements in mental clarity, reading, conversational ability, and musical performance after brain CT scans, with limited recovery lasting weeks to months after each treatment (106). Both reports are uncontrolled and involve small sample sizes, so they cannot establish efficacy; however, the consistency of the observations across independent groups supports further investigation in adequately powered, controlled trials. In the 5xFAD studies, neuroprotective effects occurred without amyloid-β clearance, suggesting that therapeutic radiation acts primarily by suppressing neuroinflammation and preserving synaptic integrity—a mechanism distinct from both the amyloid cascade and the eCIRP-driven tau phosphorylation that mediates radiation-induced dementia. Recent systematic examinations of LDRT for AD have further characterized its therapeutic potential and limitations (107, 108). A recent interim analysis of a pilot clinical trial in five patients with mild-to-moderate AD treated with six fractions of 0.5 Gy reported neurological improvement in only one patient, with the improvement being temporary, further underscoring the need for larger controlled studies (109). A comprehensive narrative review of radiotherapy for non-cancer diseases, including AD, similarly concluded that while preclinical data are encouraging, clinical evidence remains insufficient to support therapeutic use outside of carefully designed trials (110). Together, these observations suggest that the same physical agent—ionizing radiation—can cause or mitigate AD-like pathology depending on dose, fractionation, the molecular state of the brain at the time of exposure, and the specific cellular pathways activated, underscoring the importance of mechanistic context in interpreting radiation–brain interactions across the full dose spectrum.

## 5 Structural brain changes from radiation exposures

These molecular pathways produce measurable structural and metabolic changes in the brain, particularly in the hippocampus and cerebellum.

### 5.1 Hippocampal damage

An immunohistochemical and stereological analysis of mouse hippocampus at 7 days, 3 weeks, and 2 months following 17-Gy whole-brain irradiation revealed progressive reduction in the number of neuronal nuclei (NeuN)-positive neurons in the CA1 region (at 2 months), CA3 region (at 7 days), and dentate gyrus (at 7 days, 3 weeks, and 2 months) (95). Although overall neuronal counts by nuclear morphology were unchanged, indicating that the loss of NeuN expression does not necessarily indicate complete neuronal death, this finding demonstrates that ionizing radiation causes selective loss of neuronal protein expression in hippocampal circuits critical for memory formation, which could explain cognitive dysfunction independent of wholesale neuronal death. A proton magnetic resonance spectroscopy analysis of rat brain 18–19 weeks after fractionated whole-brain irradiation (35 Gy in seven fractions) revealed significant decreases in the total N-acetylaspartate to total creatine ratio in the striatum and olfactory bulb and significant decreases in the γ-aminobutyric acid (GABA) to creatine ratio in the olfactory bulb and hippocampus (91). These metabolic alterations reflect impaired neuronal function and neurotransmitter synthesis, providing evidence that ionizing radiation induces long-term metabolic dysfunction in multiple brain regions involved in memory, emotion, and learning.

### 5.2 Cerebellar damage

A study of melatonin-protective effects against oxidative damage in mouse cerebellum following exposure to ^56^Fe particle radiation identified that irradiation augmented the number of terminal deoxynucleotidyl transferase-mediated nick end-labeling (TUNEL)-positive apoptotic cells, increased DNA migration in comet assay tail measurements indicating DNA breaks, elevated carbonyl content and lipid peroxidation markers (4-hydroxyalkenal and malondialdehyde), and reduced the count of intact Purkinje cells compared with controls (111). Cerebellar Purkinje cells are thus selectively vulnerable to radiation-induced oxidative damage and apoptosis. Their loss may contribute to motor and cognitive dysfunction, fitting the broader pattern of radiation-induced neurodegeneration.

## 6 Clinical implications and evidence gaps

These structural and metabolic changes accumulate over time, shaping the clinical trajectory from radiation exposure to cognitive decline.

### 6.1 Radiation-dementia timeline and progression

Available evidence suggests that ionizing radiation can induce a temporal sequence of molecular and cellular changes leading to cognitive decline: within hours to days, gene expression changes and microglial activation occur; within days to weeks, synaptic damage, tau phosphorylation, and neuronal loss progress; within months, measurable cognitive deficits appear; and within years, a progressive dementia-like syndrome develops (91, 92, 98, 102). This extended timeline helps explain why the radiation–dementia link has been hard to establish epidemiologically: the latency between exposure and clinical dementia can span decades, complicating causal attribution.

### 6.2 Age-dependent vulnerability

A study examining the effects of mild hypothermia and hyperthermia applied for 4 h after single-dose irradiation (8 Gy) to the developing brain of postnatal day 9 rats found that hypothermia decreased radiation-induced cellular injury markers and caspase-3 activation in the subventricular zone and dentate gyrus, whereas hyperthermia exacerbated both the acute cellular damage and the loss of neural stem cells measured at 7 days postirradiation (112). The developing brain is thus extraordinarily sensitive to radiation, and thermal modulation of this vulnerability suggests that children receiving cranial radiotherapy may face elevated risk for late-onset cognitive decline.

### 6.3 Evidence gaps on radiation as an environmental cause of AD

Despite meta-analytic evidence demonstrating an 11% risk increase per 100 mSv (10), substantial gaps persist in our understanding of radiation–dementia links in humans. These gaps include (1) limited number of included studies in the meta-analysis (n=18), constraining statistical power; (2) heterogeneous exposure characterization across studies, ranging from acute high-dose medical/occupational exposures to chronic LDIR environmental exposures, limiting dose–response precision; (3) limited incident outcome data, with most included studies reporting dementia mortality rather than incident dementia cases, potentially introducing surveillance bias and reducing observed effect sizes; (4) difficulty in controlling for potential confounders such as chemical co-exposures and lifestyle factors; (5) challenges in retrospective dosimetry for individuals with uncertain exposure histories; (6) the requirement for decades of follow-up to observe dementia development, extending beyond the follow-up period available in many occupational and environmental cohorts; (7) uncertain dose thresholds and dose–response relationships for dementia risk in humans, particularly regarding the potential for hormetic effects at low doses; (8) individual variation in genetic susceptibility (particularly APOE genotype, ATM polymorphisms) that may explain why some exposed individuals develop dementia whereas others do not; and (9) the lack of validated biomarkers to identify individuals at high risk for radiation-induced cognitive decline before clinical manifestations emerge (8, 9, 13–16, 49, 85, 92).

Future epidemiological studies should employ standardized exposure assessment protocols, prospective incident outcome documentation with neuropsychological testing, adjustment for major confounders, larger sample sizes enabling stratified analyses, and integration of genetic susceptibility markers to strengthen causal inference beyond the current meta-analytic synthesis (10). Establishing causality in human populations remains difficult despite strong mechanistic and occupational evidence. An interdisciplinary Multidisciplinary European Low Dose Initiative (MELODI) expert group reached a similar conclusion, noting evidence of cognitive effects from LDIR in both biology and epidemiology but calling for better characterization of specific cognitive outcomes across the lifespan, integration of imaging biomarkers, identification of genetic susceptibility loci, and well-conducted research in large cohorts and appropriate animal models (113). More broadly, MELODI recently highlighted the implications of radiation effects on the brain for radiation protection policy, including the classification of neurological effects as tissue reactions versus stochastic effects, the identification of target organs for dose monitoring, and the need to account for individual differences in radiation responses by sex, age, genetics, and comorbidity (96). The most ambitious effort to date to address these gaps is the Million Person Study (MPS), which links individual radiation dose reconstructions for approximately one million US radiation workers and veterans to Medicare claims data for neurocognitive diagnoses, and thus should provide unprecedented statistical power for dose–response analyses at low cumulative doses (114). The meta-analytic endpoint of all-cause dementia (10) encompasses both neurodegenerative (AD) and cerebrovascular contributions. Given radiation’s established cerebrovascular toxicity (85), vascular mechanisms likely contribute substantially to the observed risk. Notably, eCIRP may drive both components: tau phosphorylation through IL-6Rα/Cdk5 on the neurodegenerative side, as detailed in Section 2.2, and endothelial injury on the vascular side, as detailed in Section 2.4. Future epidemiological studies should distinguish dementia subtypes in radiation-exposed populations to determine the relative contributions of each mechanism and to assess whether eCIRP inhibition could address both vascular and neurodegenerative pathology simultaneously. eCIRP is elevated in cerebrospinal fluid and plasma of AD patients (41), providing direct human evidence that this pathway is active in the disease state. Measuring CSF eCIRP and phosphorylated tau in patients undergoing therapeutic cranial irradiation or in occupational cohorts with documented radiation exposure would directly test whether the eCIRP-tau pathway operates in the human brain following radiation exposure. Validating eCIRP as a CSF biomarker in these populations would advance both biomarker development and mechanistic understanding.

### 6.4 Open questions and future directions for eCIRP research

Our group established the eCIRP/IL-6Rα/Cdk5 pathway using complementary genetic (CIRP-knockout mice) and pharmacological (C23) loss-of-function approaches, with gain-of-function validation by intracerebroventricular eCIRP injection (35, 40–42). These bidirectional experiments provide strong internal validation, but independent replication is a natural and important next step. The upstream DAMP biology of eCIRP—including TLR4- and TREM1-mediated signaling—has been broadly validated by independent investigators in sepsis, ischemia–reperfusion, atherosclerosis, and trauma models (84, 88, 115–118), but the neuronal eCIRP/IL-6Rα/Cdk5 cascade and its link to radiation-induced tau phosphorylation have been established exclusively by our group. Independent testing of the eCIRP-tau axis in the radiation and AD contexts specifically remains an important priority for the field. The evidence reviewed above supports eCIRP as a convergent mediator linking radiation to Alzheimer’s-like tau pathology, but important questions remain. How radiation triggers eCIRP release from brain cells is not fully understood. DNA damage and oxidative stress promote nuclear-to-cytoplasmic CIRP translocation, but the signaling intermediaries are poorly defined, and whether active secretion or passive release from dying cells predominates is unknown. The effects of low-dose and chronic radiation exposure on eCIRP levels and downstream tau pathology also remain largely unexplored. Most mechanistic studies have used acute high-dose exposures, whereas real-world exposures are chronic and low-dose. Whether eCIRP is induced at lower doses—and whether the hormetic pattern applies to eCIRP release—is a critical gap.

Although the eCIRP/IL-6Rα/Cdk5 pathway has been established as a driver of radiation-induced tau hyperphosphorylation (35, 40, 42), the extent to which eCIRP also activates other tau kinases—including GSK3β, JNK, and ERK—has not been systematically examined. A closely related question is how eCIRP-driven tau pathology relates to the canonical amyloid-β cascade. It remains to be determined whether radiation-induced eCIRP/tau phosphorylation occurs independently of amyloid-β accumulation or requires amyloid-β as a co-trigger. The acute radiation model demonstrates tau hyperphosphorylation on a timescale of hours to days that likely precedes amyloid plaque formation, suggesting that eCIRP may drive tau pathology through an amyloid-independent pathway, but this has not been formally established. It is also unknown whether eCIRP influences APP processing or amyloid-β clearance. Given that ATM dysfunction is known to drive APP accumulation (55, 58, 59), and that eCIRP is itself induced by amyloid-β in a positive feedback loop (40), eCIRP may participate in both the upstream generation and downstream consequences of amyloid pathology through mechanisms that remain to be characterized. How the eCIRP pathway interacts with the canonical amyloid-then-tau sequence in sporadic AD also deserves investigation. eCIRP may accelerate tau pathology independently of amyloid, interact synergistically with the amyloid cascade, or bridge amyloid-induced microglial activation and downstream tau hyperphosphorylation.

Beyond IL-6Rα, eCIRP is also known to activate the TLR4/MD2 complex as well as TREM1 (36), but the functional relevance of these receptors for eCIRP’s role in the development of AD remains to be fully characterized. Whether eCIRP-induced mtDNA fragmentation through TLR4-MyD88 signaling (81) occurs in irradiated brain microglia and whether the resulting cytosolic mtDNA activates the cGAS-STING innate immune pathway to amplify neuroinflammation are testable questions that would connect eCIRP biology to an established driver of sterile neuroinflammation in neurodegeneration. The cell-type specificity of eCIRP’s neuroinflammatory effects in the brain also warrants further investigation. Microglial and neuronal responses to eCIRP have been partially characterized. While eCIRP-mediated endothelial activation and PANoptosis have been demonstrated in peripheral vascular beds (72, 87), whether these mechanisms operate at the BBB in the irradiated brain requires direct investigation. The effects of eCIRP on astrocytes and oligodendrocytes, and their contributions to radiation-induced cognitive dysfunction, also remain incompletely defined. Whether eCIRP’s role extends beyond radiation to other environmental causes of AD is an open question. Its induction by ischemia, alcohol, and amyloid-β (37, 38, 40) suggests it could be a shared mediator linking cerebrovascular disease, alcohol-related neurodegeneration, and the chronic inflammation of sporadic AD. Sex differences in eCIRP signaling across these cell types—including whether estrogen modulates microglial eCIRP release or neuronal IL-6Rα sensitivity—have not been examined and may contribute to the well-documented female predominance in AD incidence. Finally, whether acute eCIRP elevation leads to persistent tau pathology and progressive neurodegeneration, or whether compensatory mechanisms resolve the insult, is a fundamental unanswered question—one directly relevant to the decades-long latency between radiation exposure and clinical dementia. Addressing these questions is essential for validating eCIRP as a therapeutic target and translating inhibitor strategies to the clinic. Translating eCIRP inhibition from preclinical proof-of-concept to clinical application requires addressing several tractable challenges: optimizing CNS delivery (C23 BBB penetration, alternative small-molecule eCIRP inhibitors, or intrathecal delivery strategies); characterizing pharmacokinetics and safety in chronic dosing models; and demonstrating efficacy in transgenic AD models with progressive pathology. Given that eCIRP activates multiple pathological cascades, its inhibition offers multipathway intervention through a single target—unlike kinase-selective or amyloid-specific approaches. Development of CSF eCIRP as a companion biomarker would enable patient stratification for clinical trials and treatment response monitoring.

### 6.5 Sex differences in radiation sensitivity and AD risk

Sex is an important but understudied variable in radiation-AD risk. Women account for approximately two-thirds of all AD cases—a disproportion exceeding what would be expected from differences in longevity alone—whereas biological sex is also known to modify radiation sensitivity across multiple health outcomes. Together, these observations raise important questions about radiation-induced dementia risk. Notably, the Ohtaki et al. analysis of non-initial radiation exposure in Hiroshima survivors found substantially higher excess relative risks for solid cancer mortality in men than in women in downwind districts (50), yet women bear the greater burden of sporadic AD—suggesting that the direction of sex-dependent radiation sensitivity may differ by endpoint and that radiation-induced dementia risk may not follow the same sex distribution as radiation-induced cancer risk. At the molecular level, estrogen exerts well-characterized neuroprotective and anti-inflammatory effects in the central nervous system, including suppression of microglial activation and modulation of tau phosphorylation through Cdk5 and GSK3β (119–121), which could buffer premenopausal women against radiation-induced eCIRP-mediated tau pathology. A comprehensive review has detailed how estrogen modulates three hallmarks of radiation-induced neurodegeneration—oxidative stress, neuroinflammation, and impaired neurogenesis—and highlighted phytoestrogens as potential adjuvant neuroprotectants during radiotherapy (122). Whether eCIRP release, IL-6Rα expression, or p25/Cdk5 activity differ by sex under radiation-stressed conditions has not been examined and represents an important gap. Postmenopausal loss of estrogen-mediated neuroprotection, combined with cumulative lifetime radiation exposure, could contribute to the disproportionate AD burden in older women (123). Future studies examining radiation–dementia associations should stratify by sex and menopausal status, and preclinical studies should examine whether sex modifies eCIRP release or downstream tau kinase activity following radiation exposure.

## 7 Summary: the radiation–dementia causal chain

The evidence reviewed above supports a pathogenetic chain connecting ionizing radiation to Alzheimer’s-like neurodegeneration through DNA damage, oxidative stress, and eCIRP-mediated neuroinflammation. The initial radiation insult generates DNA double-strand breaks and excessive reactive oxygen species that directly activate the ATM kinase and trigger rapid microglial activation within hours of exposure, leading to nuclear-to-cytoplasmic translocation and release of eCIRP (**Figure 2A**). Released eCIRP activates the neuronal IL-6Rα/PLC/IP3/calpain/p25/Cdk5 cascade (detailed in Section 2.2), driving pathological tau phosphorylation at Ser199, Ser202, and Ser396—the same sites found in AD neurofibrillary tangles (**Figure 2B**) (35, 41).

Beyond this established pathway, eCIRP likely contributes to cognitive impairment through additional mechanisms including neuroinflammatory amplification, A1 astrocyte induction, mitochondrial dysfunction and ferroptosis in myeloid cells (82), blood–brain barrier disruption through endothelial activation and PANoptosis (72, 87), suppression of neurogenesis, synaptic dysfunction, and impaired amyloid-β clearance (**Figure 3**). Lactate accumulation from radiation-induced glycolytic reprogramming may further amplify eCIRP release through CIRP lactylation (84), establishing a metabolic feedforward loop that sustains neuroinflammation beyond the initial insult. Simultaneously, radiation-induced ATM activation and impaired ATM signaling in vulnerable neuronal populations promote aberrant cell cycle reentry and apoptotic death through p53-dependent mechanisms, whereas age-dependent decline in ATM function with cumulative radiation exposure progressively removes protection against DNA damage-induced neuronal death and permits accumulation of genomic instability that further drives amyloid precursor protein expression (55, 59). The eCIRP/IL-6Rα/Cdk5-mediated tau phosphorylation cascade is amplified through positive feedback mechanisms where eCIRP itself promotes additional microglial activation and eCIRP release, creating a self-perpetuating neuroinflammatory state that drives sustained tau hyperphosphorylation, PHF aggregation, synaptic dysfunction, and ultimately the hippocampal and cortical neuronal loss underlying radiation-induced cognitive decline.

**Figure 3:**
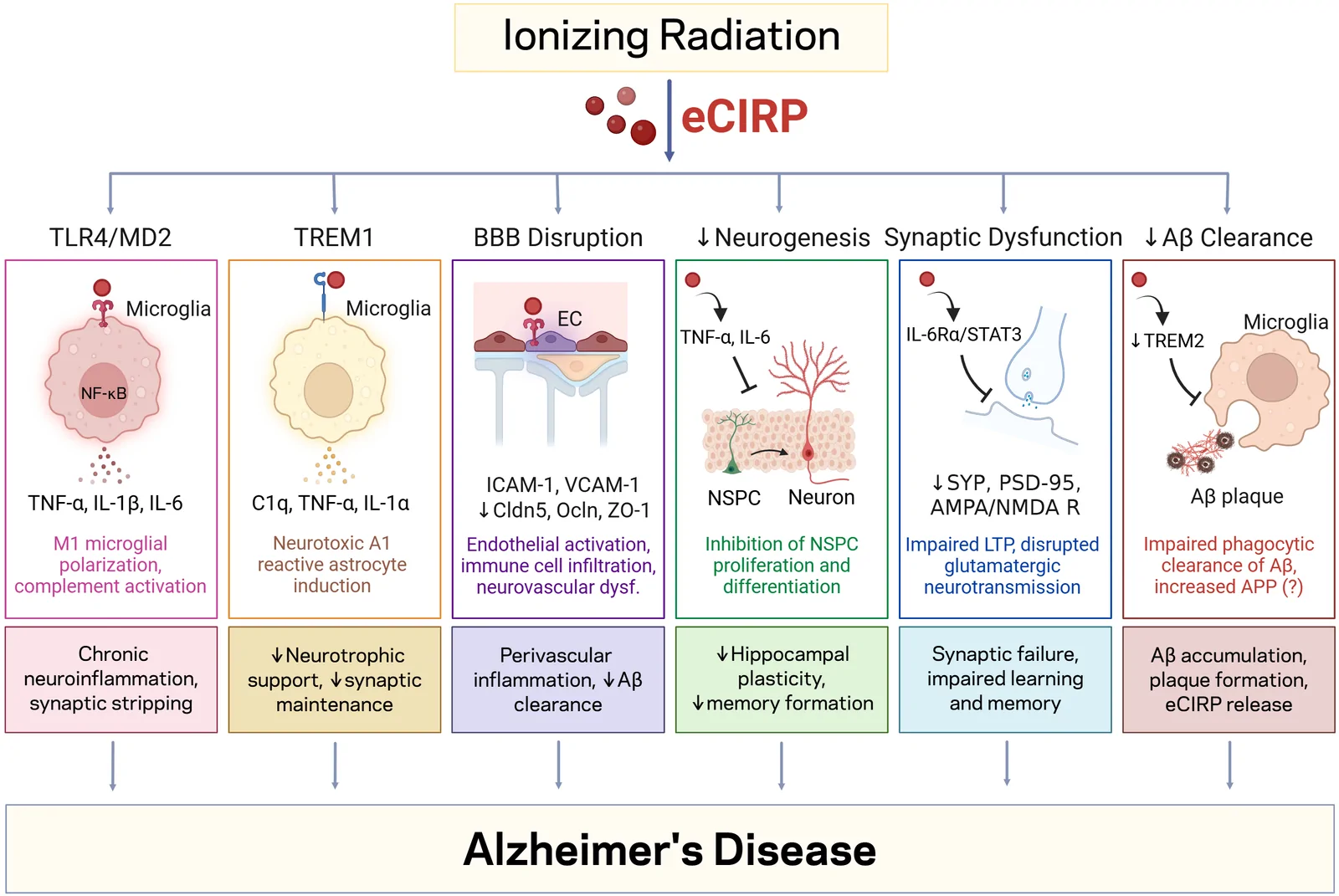
Hypothesized additional eCIRP-mediated mechanisms contributing to radiation-induced AD beyond direct tau phosphorylation. Beyond the experimentally established eCIRP/IL-6Rα/Cdk5 tau phosphorylation cascade (**Figure 2**), radiation-induced eCIRP is hypothesized to drive AD pathology through six additional mechanisms based on known eCIRP signaling in non-neural systems: (1) TLR4/MD2-mediated neuroinflammatory amplification via M1 microglial polarization; (2) TREM1-mediated neurotoxic A1 astrocyte induction; (3) blood–brain barrier disruption through endothelial TREM1 activation and neutrophil-mediated PANoptosis; (4) suppression of hippocampal neurogenesis by proinflammatory cytokines; (5) synaptic dysfunction via IL-6Rα/STAT3 signaling; and (6) impaired Aβ clearance through TREM2 downregulation and possible ATM-mediated APP upregulation (indicated by question mark). All six pathways are proposed to converge on progressive AD pathology. These mechanisms are extrapolated from peripheral eCIRP biology and established radiation neurobiology; direct validation in the irradiated brain remains to be performed. See Sections 2.3–2.5 for detailed mechanistic descriptions. Abbreviations are defined at first use in the main text. References (35, 36, 40, 41, 55, 58, 59, 67, 72, 73, 79–83, 85, 87–89, 91, 92).

This pathogenetic chain is therapeutically modifiable at multiple points. ATM inhibition paradoxically reduces some downstream neurotoxic effects, ATM activation through LDIR hormesis may suppress inflammation and tau pathology, and critically, eCIRP inhibition through the C23 peptide abrogates radiation-induced tau phosphorylation, establishing eCIRP as a convergent therapeutic target and suggesting that eCIRP inhibitors deserve investigation for both radiation-induced cognitive dysfunction and sporadic AD (35, 41).

## 8 Conclusion

Ionizing radiation is associated with increased dementia risk. Mechanistic evidence identifies eCIRP-mediated neuroinflammation as a convergent pathway that drives radiation-induced tau pathology through IL-6Rα/Cdk5 signaling. This pathway is validated by genetic and pharmacological loss-of-function studies demonstrating marked attenuation of radiation-induced tau phosphorylation *in vivo*. Beyond direct tau phosphorylation, eCIRP likely drives additional pathological processes including neuroinflammatory amplification, A1 astrocyte neurotoxicity, mitochondrial dysfunction and ferroptosis, blood–brain barrier disruption via endothelial TREM1 activation and neutrophil-mediated endothelial PANoptosis, suppression of hippocampal neurogenesis, synaptic dysfunction, and impaired amyloid-β clearance, positioning eCIRP as a central node in radiation-induced neurodegeneration. Key priorities for advancing this framework include dose–response characterization of eCIRP release at occupationally relevant doses, demonstration of cognitive and behavioral consequences of eCIRP-mediated tau phosphorylation, testing of eCIRP inhibition in chronic AD models, and measurement of eCIRP in human cerebrospinal fluid following radiation exposure. eCIRP inhibitors warrant investigation for both radiation-exposed populations and patients with sporadic AD, where eCIRP is similarly elevated. Translating eCIRP-targeted therapies to the clinic, guided by eCIRP biomarker development, offers a promising direction for addressing both radiation-induced and age-related cognitive decline.
